# News and Events of the Week

**Published:** 1910-03-19

**Authors:** 


### News and Events of the Week.

A Bill to amend the law relating to the tenure of office of parochial medical officers in Scotland has been presented by Mr. C. Wason, M.P. for Orkney and Shetland.

The London Ophthalmic Exhibition will be held at the rooms of the Medical Society of London on Friday, March 11 and 12, from noon to 9 P.M. The conferences announced for to-day have had to be postponed.

The German Röntgen Society will hold its sixth congress in Berlin, beginning on April 3, 1910, at the Langenbeckhaus. Tickets and further particulars may be obtained on application to the Secretary, Dr. Immelmann, Berlin, W. 35, Lützowstrasse 72.

Sir Alfred Keogh, K.C.B., has accepted the Rectorship of the Imperial College of Science and Technology, and will take up his work at an early date. He retired last year from the post of Director-General of the Army Medical Service, which he had held from 1905.

"Typhoid Mary," the woman who a few years ago was stated to be a "typhoid carrier," and the cause of a series of local epidemics of typhoid fever, and who has been quarantined for three years on North Brother's Island in New York, has finally been released by the Board of Health on the condition that she will not return to her former occupation of a cook, and will report to the Board of Health at frequent intervals.

A man about 50 years of age, who was recently found dead in one of the lavatories at Paddington Station with an artery in his wrist severed and a surgeon's knife on the floor, showing that he had bled to death, has been identified as Dr. Charles Nugent Fox, and papers found in the pockets gave an address near Cardiff.

Dresden International Hygiene Exhibition, 1911.—At a meeting of members of the British Executive Committee of this Exhibition, held on Tuesday at the Hotel Cecil, Professor Dr. Pannwitz, the deputed representative of the Scientific Department of the Exhibition, delivered an address. Among the ladies and gentlemen present were Mr. B. A. Whitelegge, H.M. Chief Inspector of Factories (Home Office); Mr. H. R. Kenwood, Professor of Hygiene at University College; Sir John Purley, of the St. John Ambulance Association; Mr. J. Porter, Director-General of the Medical Department of the Royal Navy, and many others. Dr. Pannwitz explained at considerable length the aims and objects of the Exhibition, the support which was being so liberally extended by the German Imperial and State Governments, the vigorous efforts which every civilised country was making to secure an effective representation, and concluded by expressing his full confidence that the British representation would be in every respect worthy of the country which was the acknowledged birthplace of sanitary science. The speech was very warmly received, and after various other speeches had been delivered by Dr. Williams, Major Pollock, Dr. Armit, Sir John Purley, Mr. Whitelegge, and others in approval of the project, and offering many practical suggestions, the proceedings concluded. Offices will be opened in Victoria Street, S.W., for the accommodation of the British Executive and for the general working of the undertaking in this country.

A deputation representing the Toronto Academy of Medicine waited on the Cabinet of the Ontario Legislature, on February 14, and requested that a Pasteur Institute be established in Toronto, in connection with the University. In view of the fact that there is quite a "mad dog scare" in the western part of Ontario at the present time, and that many victims of dog bites have gone to the Pasteur Institute in New York for treatment, the physicians of Toronto contend that such an institute should be established as soon as possible.

The report of Dr. F. W. Pavy, F.R.S., principal delegate of his Majesty's Government to the International Congress of Medicine held at Budapest from August 29 to September 4, 1909, has been issued as a Parliamentary Paper [Cd. 5047]. The next Congress will be held in 1913 in London. There has been hitherto no direct connecting link between one Congress and another. To escape from this broken continuity the executive committee of the Budapest Congress suggested the creation of a "Permanent International Commission," which was agreed to, and rules were framed for its course of action. Dr. Pavy was appointed President of the Commission, and The Hague was fixed on for its bureau.

Recognition to Professor John Cleland, M.D., LL.D., D.Sc., F.R.S.—An Executive Committee was appointed some time ago to arrange for some appropriate acknowledgment of the long and distinguished services of Professor John Cleland in connection with the University of Glasgow. The committee may be congratulated on having obtained the consent of Sir George Reid, R.S.A., to undertake the work, which will be greatly facilitated by the happy circumstance that both the artist and the sitter are now permanently settled in Somerset within a few miles of each other. The committee in charge of the arrangements have ascertained that this proposal meets with the approval of the distinguished professor. Of course, some time must necessarily elapse before the ceremony can take place, but due notice will be sent to subscribers.

Speaking on tropical diseases in the House of Commons, Sir W. Collins asked the attention of the Committee to the vote of £1,000 for Entomological Research (Tropical Diseases). This vote, he said, was a new departure on the part of the Colonial Office, and one that he hoped would be supported on all sides of the House. He wished to know whether it was proposed to include in the investigation, not only the question of the diseases of man, but also the diseases of animals, including the African horse fever. He hoped that the Commission would take a wide view of the natural history of disease, and not merely make laboratory experiments. Mr. Churchill, in reply, said the Committee over which Lord Cromer presided was purely a committee of research, and was separate and independent altogether of the Liverpool and London Schools of Tropical Medicine. The inquiry covered the whole region of the diseases which were conveyed by insects, not only insects which infected man, but animals and plants as well. He could not possibly make any pledge to cut the Committee off from any line of inquiry they thought to be desirable in minimising animals' sufferings so far as science was not impeded. Dr. Addison urged that the Committee should not be tied in any way, and that there should be co-ordination between the efforts made in different parts of the world in reference to these diseases. He called attention to smallness of the vote, which was agreed to.

